# The Engineering Side of Hospital Work

**Published:** 1905-09-16

**Authors:** 


### THE ENGINEERING SIDE OF HOSPITAL WORK.

### XIV. ELECTRICAL APPARATUS.

(*Continued from page* 194.)

#### The Increase or Decrease of Osmotic Pressure.

Osmotic pressure is that exerted by the vapour that is emanating from a fluid, upon the surface of the fluid. It is also the pressure exerted by each of two solutions present in a vessel, tending to cause mixture. When the two solutions are separated by a porous diaphragm, the osmotic pressure is that which is exerted by the solutions upon the diaphragm. Where, as in so many of the physiological operations that take place within the body, the porous diaphragm has a selective function, allowing only the passage of certain substances, the pressure of the substances that can pass is the osmotic pressure, and it is this pressure that the electric current will assist, or oppose. When a current of electricity passes through a porous membrane, it tends to carry the liquid by which it is passing with it, through the membrane, and so to accelerate its rate of passage. The acceleration is approximately in proportion to the strength of the current. A familiar instance of this is the fact that in galvanic batteries, where a porous cell is employed, the passage of the current materially aids the cell in acquiring its full strength, by accelerating the passage of the liquid to the negative plate.

#### Mechanical Action of Electric Discharge.

The spark discharge has a mechanical action somewhat similar to that described in connection with osmotic pressure. It carries portions of the substance of the positive conductor towards the negative, and it also mechanically disrupts insulating substances, such as cardboard, glass, etc., that may be in its path. The wearing away of the positive carbon of arc lamps and of the positive side of switches is due to the first property, and some of the violent effects of lightning and high tension currents are due to the second.

#### Electro-magnetism.

The well-known fact that when a current is sent round a piece of iron the iron becomes magnetic is not what the writer wishes to refer to, though it is the explanation of the action of the telephone, the dynamo, and other apparatus. What he wishes to refer to is—whenever an electric pressure is applied to a conductor, as the resulting current passes through the conductor, a magnetic field is created round the conductor, and this field must be created right along the conductor before the current can arrive at the end. When the current ceases in the conductor the reverse operation takes place, the energy that was expended in creating the magnetic field around the conductor returns to it and creates an electric pressure in the conductor, in the same sense as that which caused the first current. Also, when any change takes place in the strength of the current passing, an equivalent change is caused in the magnetic field surrounding the conductor, lessening the current causing the temporary creation of a pressure in the same direction as the primary pressure, increase in the current strength causing the creation of a pressure in opposition to it. Also the rise or fall of a current in one conductor creates a pressure in every other conductor in the neighbourhood, in such a direction that the current resulting tends to resist the rise or fall of the primary current, the resistance offered being in the form of a secondary magnetic field, whose operation will be such as to create a pressure in the primary conductor tending to resist the change. Further, the motion of a conductor in a magnetic field creates a pressure in itself, tending to resist the motion, the resistance being offered by way of the secondary magnetic field, and the currents created by it. These properties are of great importance in connection with alternate currents, and also in connection with currents passing in the body, inasmuch as in every part of the body there are blood-vessels in which the blood is moving in opposite directions, and as the blood-vessels act as conductors when a current is passing through the part of the body in which they are situated, the motion of the blood must have a considerable effect upon the action of the currents.

#### Forms of Electricity.

As mentioned above, there is only one electricity, but it is presented in many different forms. Those with which we are here concerned are:—

Electricity generated by static, or frictional machines, in which plates of glass or vulcanite are revolved.

Continuous electric currents, generated by batteries or dynamos.

Alternate currents generated by dynamos, and of these there are:—Single-phase currents, or as the French electricians call them simple alternate currents; two-phase currents, and three-phase currents; high-frequency currents.

There are also subsidiary forms of currents such as those delivered by induction coils, interrupted currents, and others.

Differences Between Continuous and Alternate Currents.

Certain important differences between continuous and alternate currents should be carefully noted.

Continuous currents are those known as constant currents in medical works. Alternate currents are usually referred to in medical works as sinusoidal currents. Both terms are incorrect. A continuous current may be constant for a time, but that is not its characteristic feature. An alternate current may be of such a nature that its characteristic curve is sinusoidal in form, but again that is not its principal feature. The sharply-defined distinction between the continuous current and the alternate current lies in the form in which each is generated. The continuous current is a continuous procession of waves, adopting the wave theory, all in one direction, and the currents are of the same strength, as long as the resistances and pressures opposed to them are the same. The alternate current is not only alternating in direction; it is constantly changing in value. Thus, with the alternate currents that are used for lighting and power in electricity supply stations, the pressures are constantly rising and falling, 100 times a second, and alternating 50 times. The frequency, as it is termed, now usually employed in supply services is 50 periods per second. That means that in every fiftieth of a second the pressure rises from zero to a maximum considerably above the working pressure, falls to zero, rises to a maximum in the opposite direction, and falls to zero again. With high-frequency currents, the alternations take place 1,000,000 times a second. The point, however, that is important in connection with this great difference between the two forms of current is the effect which the continual changes have upon the magnetic fields and the secondary effects of the changes in the magnetic fields upon the currents passing in the conductors themselves. Each change in the pressure, as explained, causes a change in the magnetic field, which reacts upon the current passing in the conductor. One result of this is, the currents and the pressures with alternate current services, do not act together, the current is always behind the pressure. This sounds somewhat odd, but the explanation is, the changes in the pressure and in the current create pressures tending to delay the passage of the current, just as it is delayed with continuous currents when the pressure is first applied. The effect reaches farther than that, however. It leads to the alternate current not being able to make use of the inner portions of a conductor. For this purpose, a solid copper rod say, must be looked upon as a mass of infinitely small rods in a bundle, and when a current rises in one rod, pressures are created in all the other rods, in such a manner that the passage of the current in the first rod is resisted and delayed. As this happens to all the rods, each exerting its effect upon the current passing in all the rest, the result is the current has not time to penetrate the mass of the conductor. With the ordinary alternate current services only about three-fifths of the rod take part in the action of conveying the current. For this reason, in certain cases, tubes are used as conductors. With alternations of the high-frequency type, 1,000,000 per second, it will easily be seen, following the same reasoning as above, that the currents cannot penetrate the surface of the body, and are actually carried over the surface of the skin; hence the ability to use pressures of the order of 1,000,000, not only without danger, but with benefit. It should be mentioned that the lightning discharge is usually in the form of a high-frequency current, the discharge being oscillating, not continuous, the action being somewhat like that of the pendulum. High-frequency currents may therefore be obtained from static electric apparatus, by suitable arrangement, as will be explained.

The phenomena referred to above have also an important bearing upon the passage of alternate currents, and of currents whose value is changing, through the tissues in the body, the blood moving in the blood-vessels serving to add to the effect.

(*To be continued.*)

